# Characterization of vB_StuS_MMDA13, a Newly Discovered Bacteriophage Infecting the Agar-Degrading Species *Sphingomonas turrisvirgatae*

**DOI:** 10.3390/v12080894

**Published:** 2020-08-15

**Authors:** Pasquale Marmo, Maria Cristina Thaller, Gustavo Di Lallo, Lucia Henrici De Angelis, Noemi Poerio, Federica De Santis, Maurizio Fraziano, Luciana Migliore, Marco Maria D’Andrea

**Affiliations:** 1 Department of Biology, Tor Vergata University, 00133 Rome, Italy; pasquale.marmo@uniroma2.it (P.M.); thaller@uniroma2.it (M.C.T.); dilallo@uniroma2.it (G.D.L.); lucia.henrici@gmail.com (L.H.D.A.); noemi.poerio@gmail.com (N.P.); federica.desa92@gmail.com (F.D.S.); fraziano@bio.uniroma2.it (M.F.); luciana.migliore@uniroma2.it (L.M.); 2 Department of Medical Biotechnologies, University of Siena, 53100 Sienna, Italy

**Keywords:** bacteriophage, *Siphoviridae*, *Sphingomonas* spp.

## Abstract

Members of *Sphingomonas* genus have gained a notable interest for their use in a wide range of biotechnological applications, ranging from bioremediation to the production of valuable compounds of industrial interest. To date, knowledge on phages targeting *Sphingomonas* spp. are still scarce. Here, we describe and characterize a lytic bacteriophage, named vB_StuS_MMDA13, able to infect the *Sphingomonas turrisvirgatae* MCT13 type strain. Physiological characterization demonstrated that vB_StuS_MMDA13 has a narrow host range, a long latency period, a low burst size, and it is overall stable to both temperature and pH variations. The phage has a double-stranded DNA genome of 63,743 bp, with 89 open reading frames arranged in two opposite arms separated by a 1186 bp non-coding region and shows a very low global similarity to any other known phages. Interestingly, vB_StuS_MMDA13 is endowed with an original nucleotide modification biosynthetic gene cluster, which greatly differs from those of its most closely related phages of the *Nipunavirus* genus. vB_StuS_MMDA13 is the first characterized lytic bacteriophage of the *Siphoviridae* family infecting members of the *Sphingomonas* genus.

## 1. Introduction

Bacteriophages, or simply phages, are obligatory intracellular parasites able to exclusively infect bacterial cells. These prokaryotic viruses are considered the most abundant entities in the biosphere [1] and have been found in almost all the settings which support bacterial growth, from terrestrial to aquatic environments [2,3]. Phage particles are one of the primary drivers of bacterial adaptive evolution, given their major direct role in the control of the type and balance of bacterial populations, and their ability to transfer genetic material from one bacterium to another via transduction. At the same time, phages or selected products of their genomes have been largely exploited in many biotechnological applications, including food bio-preservation [4], diagnostics and therapeutics [5,6], delivery vehicles [7], molecular biology [8], disinfection of medical devices [9], and biocontrol of several eukaryotic bacterial pathogens [10]. Being viruses, phages are characterized by a huge genomic diversity, which is recently becoming more apparent, especially thanks to the advancements and low costs of high-throughput DNA sequencing techniques. This high genomic heterogeneity, that is basically due to vertical and horizontal gene transfer from other phages or microorganisms, leads in several cases to a genome mosaicism originated from recombination events mediated either by host- or phage-encoded recombination machineries [11]. Roughly 88% of the fully sequenced phage genomes deposited in publicly available DNA databases belong to the *Caudovirales* order (https://www.ncbi.nlm.nih.gov/genome/browse, last accessed on 22 July 2020), which historically represents the most well-studied and characterized group of phages [12]. This order, originally composed of three families (*Myoviridae*, *Siphoviridae* and *Podoviridae*), has been recently revised and currently includes six additional families: *Ackermannviridae*, *Autographiviridae*, *Chaseviridae*, *Demerecviridae*, *Drexlerviridae* and *Herelleviridae* [13,14,15], thus underscoring the fast changes in phage taxonomy, primarily due to the rapid accumulation of phage genomes. 

Members of the *Sphingomonas* genus have recently gained particular biotechnological interest for their prominent ability to degrade or transform a wide variety of recalcitrant compounds, including chemical contaminants produced by industrial processes, dibenzofurans or polycyclic aromatic hydrocarbons (PAH), and other noxious compounds such as insecticides, herbicides, and heavy metals [16]. These metabolic activities have been exploited either in several types of bioreactors or in *in situ* bioremediation experiments [17,18,19,20,21]. However, studies on phages targeting members of the *Sphingomonadaceae* family, and on the evaluation of their impact in bioreactors or environments bioaugmented with these species, are limited [22,23,24,25]. In particular, only three phages, i.e., a lysogenic *Siphoviridae* active against *Erythrobacter* sp. [22], a lytic *Siphoviridae* infecting *Sphingobium* sp. IP1 [24] and a *Microviridae* specific to *Citromicrobium* spp. [25] have been fully characterized. To date, only a few phages targeting *Sphingomonas* spp. have been described [26,27], and just five have been fully sequenced (Accession numbers: NC_019521.1, MH684921.1, MN734437.1, MN734438.1 and MN734439.1). These phages infect *Sphingomonas paucimobilis*, the type species of the genus, or unclassified *Sphingomonas* spp., and belong to the *Siphoviridae*, *Myoviridae* and *Autographiviridae* families, but no information is available on their lytic or lysogenic nature.

In this work we describe a newly discovered lytic phage of the *Siphoviridae* family, able to infect the *Sphingomonas turrisvirgatae* MCT13 type strain, and which is characterized by a genome having overall a very limited similarity to any other known phages.

## 2. Materials and Methods

### 2.1. Bacterial Host and Growth Conditions

The recently described agarolytic species *S. turrisvirgatae* (type strain MCT13 = DSM 105457^T^ = BAC RE RSCIC 7^T^), isolated from a drainage ditch within a disused system of constructed wetlands located in Rome [28], was used as host for the isolation and propagation of the vB_StuS_MMDA13 bacteriophage. SM buffer (10 mM Tris-HCl, pH 7.5; 100 mM NaCl; 10 mM MgSO_4_) was employed for suspending and titrating bacteria and phages. *S. turrisvirgatae* MCT13^T^ was routinely cultured in ZoBell Broth (ZBB) [29] or on plates composed by ZBB solidified with 15 g/L of agar (ZBA). Soft-agar was prepared by dissolving agar in ZBB broth to a final concentration of 0.8%. For double-layer plating an overnight culture (O/N) of the indicator strain concentrated 1/10 in SM buffer was mixed with soft-agar and aliquots of samples under analysis, as specified below. Culture media were purchased from Oxoid (Oxoid, Hampshire, UK), while chemical reagents were obtained from Sigma-Aldrich (Sigma-Aldrich, Milan, Italy).

### 2.2. Isolation, Purification and Large-Scale Production of Bacteriophage

The phage vB_StuS_MMDA13 was isolated from fresh surface waters, collected in a pond near Viterbo (Italy), by using the double-layer agar technique and MCT13^T^ as the indicator strain. Briefly, a 40 mL sample of freshwaters was centrifuged at 4,150× *g* for 10 min at 25 °C. The supernatant was filtered through a 0.2 μm syringe filter (Sarstedt, Nümbrecht, Germany), then a 1 mL aliquot was used for double-layer plating by using *S. turrisvirgatae* MCT13^T^ as indicator strain. Pure bacteriophage suspensions were obtained by three rounds of single plaque picking and re-infection of the same strain. Large-scale production of vB_StuS_MMDA13 was performed as previously described [30]. For long-term storage, phage suspensions in SM buffer were supplemented with 25% glycerol and 2% chloroform and stored at −60 °C. 

### 2.3. Transmission Electron Microscopy (TEM)

A preparation of phage particles was centrifuged at 41,200× *g* for 120 min at 4 °C in a Beckman JA-20 rotor (Beckman Coulter, Fullerton, CA, USA) and suspended at about 10^12^ PFU/mL in SM buffer; 10 μL of the concentrated phage suspension were let to adsorb on a carbon-coated matrix and then stained with 2% uranyl acetate for 15 s. The grid was subsequently washed twice with ddH_2_O, air dried and observed by a FEI Tecnai 12 (FEI, Eindhoven, The Netherlands) TEM fitted with an Osis Morada 2X4 K CCD camera (Olympus, Shinjuku, Tokyo, Japan). 

### 2.4. Determination of Bacteriophage Host Range

The host range of vB_StuS_MMDA13 was determined by the spot test technique using a total of 21 characterized strains (Table 1). The strains, available from our Laboratory collection or purchased from DSMZ (DSMZ, Braunschweig, Germany), were grown in 10 mL of different media at 30 °C with shaking to an OD_600_ of 0.5, centrifuged at 4,150× *g* for 10 min and suspended in a 1/10 of SM buffer. An aliquot of 0.2 mL of each bacterial suspension was mixed with 4.5 mL of molten soft-agar and poured onto ZBA, plate count agar (DSM 1244), nutrient agar (DSM 8826, DSM 30196 and DSM 7098), tryptic soy agar (DSM 7462 and DSM 13593), or SPhingo agar (DSM 25525) plates. Finally, a 0.01 mL volume of a phage suspension (≈10^7^ PFU/mL) was spotted on the overlay. Sensitivity to the vB_StuS_MMDA13 infection was tentatively assessed by the presence of a lysis halo at the spot after O/N or 72 h (DSM 25525) incubation at 30 ± 2 °C.

### 2.5. One-Step Growth Curve

The host strain MCT13^T^ was grown in aerobic conditions at 30 °C to mid-exponential phase (OD_600_ = 0.3–0.4), then an aliquot of 0.9 mL was infected with 0.1 mL of 1 × 10^7^ PFU/mL phage stock to arrive at a Multiplicity Of Infection (MOI) of 0.01. After 10 min at 30 °C, the mixture was centrifuged twice at 12,000× *g* for 2 min at 4 °C to remove the non-absorbed phages. The pellet was finally suspended in 1 mL of SM buffer, diluted 1 × 10^−3^ in 30 mL of ZBB and incubated at 30 °C with shaking. Aliquots of 0.1 mL were taken at 30 min intervals and phage titer was determined by double-layer agar technique. The experiment was carried out in triplicate. The latency period was computed excluding the 10 min of phage adsorption and the 5 min centrifugation interval needed to remove non-absorbed phages. The burst size was computed as the ratio of the average phage titer after the rise period to the average number of infected bacterial cells during the latency period [44,45].

### 2.6. Influence of pH and Temperature on Phage Viability

The stability of vB_StuS_MMDA13 to pH was evaluated by diluting phage particles to approximately 2.5 × 10^9^ PFU/mL in 1 mL of SM buffer, previously adjusted with 1 M NaOH or 1 M HCl to obtain a pH range from 2.0 to 12.0 with intervals of 1 unit. Phage suspensions were incubated for 60 min at 25 °C and then titrated by using the double-layer-agar method. The effect of temperature on phage viability was assessed by incubating 1 mL of phage suspension (≈2.5 × 10^9^ PFU/mL) at 25, 40, 50, 60, 70 and 80 °C for 60 min and then titrated as reported for the pH test. Both assays were carried out in triplicate and the reported values are the mean of phage counts (PFU/mL) ± standard deviation. 

### 2.7. Lysogeny Tests

A bacterial culture of *S. turrisvirgatae* MCT13^T^ grown to mid-exponential phase (OD_600_ = 0.3) was infected with vB_StuS_MMDA13 at a MOI ≈ 1,000. After 120 min from infection, an aliquot of 0.1 mL was plated in ZBA. Following two days of incubation at 30° C, three randomly selected colonies insensitive to phage action were isolated and then grown in ZBB at 30 °C until OD_600_ = 0.3. Mitomycin C at a final concentration of 0.1 μg/mL was finally added and growth was continued for additional 24 h. Bacteria were then treated with 2% chloroform, shaken briefly, set aside for 20 min at room temperature (RT), centrifuged at 4150× *g* for 5 min and supernatants containing putative temperate phages were collected and filtered through 0.2 μm syringe filter. Lysogeny verification was performed by spotting supernatants onto MCT13^T^ bacterial lawn to check for plaque formation. 

A PCR analysis was also performed on the same phage-insensitive derivatives, using two couples of vB_StuS_MMDA13 specific primers: MMDA13_terLF (5′-GAAGATCTGGTGGGACGACCTC-3′) and MMDA13_terLR (5′-ATCTCGTAATAGTGGTTCAGACCGTC-3′), targeting the terminase large subunit, and MMDA13_MCPF (5′-GACGCTGAGCTGAGCAACCTG-3′) and MMDA13_MCPR (5′-CAGCCAGGTATCGAACACACCC-3′), targeting the major capsid protein. 

### 2.8. Extraction of Bacteriophage vB_StuS_MMDA13 DNA

The vB_StuS_MMDA13 DNA was extracted from phage lysate (approximately 3 × 10^10^ PFU/mL) by using the Phage DNA Isolation Kit (Norgen Biotek Corp., Thorold, ON, Canada) following manufacturer instructions. At the end of the extraction procedure, an aliquot of the DNA preparation was resolved by agarose gel electrophoresis (0.75% *w*/*v*), stained with ethidium bromide (0.05 µg/mL) and visualized under UV light to check the integrity of phage genome and to have a rough estimate of its size. vB_StuS_MMDA13 DNA was quantified by using a Qubit fluorometer (Thermo Scientific, Waltham, MA, USA). DNA samples were stored at 4 °C.

### 2.9. Genome Sequencing and Bioinformatics Analysis of vB_StuS_MMDA13

The genome of vB_StuS_MMDA13 phage was sequenced with the MiSeq instrument (Illumina Inc., San Diego, CA, USA), by using a 2 × 300 paired-end approach and the Illumina Nextera XT™ kit. The obtained raw reads were assembled by using the SPAdes software v. 3.13.0 [46] in one single node. Phage genome was annotated by Rapid Annotations using Subsystems Technology (RAST) web-service (accessed on 6 March 2019) [47]. Automatic annotation was manually reviewed by BLASTP or BLASTX (both v. 2.9.0) comparison against RefSeq proteins deposited in the International Nucleotide Sequence Database Collaboration (INSDC) databases (release 231), and by HHpred (accessed on 12 December 2019) [48] and HMMER v. 3.3 [49] analyses. Cleavage sites of native proteins were predicted by using LipoP 1.0 (https://services.healthtech.dtu.dk/service.php?LipoP-1.0), while transmembrane helix predictions were performed by TMHMM 2.0 (https://services.healthtech.dtu.dk/service.php?TMHMM-2.0). The VirFam web-server was employed to identify proteins of the phage head-neck-tail module [50]. Analysis of conserved protein domains was performed by using the Conserved Domain Database (CDD) search tool available at the NCBI website (accessed on 10 March 2019) [51]. The tRNAscan-SE 2.0 [52] and ARAGORN (accessed on 10 April 2019) [53] web-tools were used to predict phage and host tRNA genes, respectively. Putative promoters and transcriptional terminators were searched with the Bprom (http://www.softberry.com/, last modification date: 24 October 2016) and the Genome2d v. 2.0 (http://genome2d.molgenrug.nl/g2d_pepper_transterm.php) tools, respectively. Phylogenetic analysis was performed by using the terminase large subunits or major capsid protein sequences of bacteriophages belonging to the *Nipunavirus*, *Yuavirus*, *Nonagvirus*, *Vidquintavirus* and *Seuratvirus* genera reported by the International Committee on Taxonomy of Viruses (ICTV) classification (https://talk.ictvonline.org/taxonomy/; last accessed on 30 July 2020). In both cases closest homologues, i.e., proteins having a >50% identity and ≥98% coverage in a BLAST search (https://blast.ncbi.nlm.nih.gov/), have also been included. Protein alignments were performed using ClustalW with default parameters in the MEGA X software [54], while the maximum likelihood method with bootstrap analysis of 100 replicates was used to build phylogenetic trees. 

The OrthoANI software v. 0.93.1 [55] was used to estimate the overall genome sequence similarity between vB_StuS_MMDA13 and the same set of phages used in the analysis of terminase large subunits and major capsid proteins. Comparison of phage genomes was also performed by using the VICTOR web-server using settings recommended for prokaryotic viruses (accessed on 22 January 2020) [56], while Easyfig [57] was employed to compare vB_StuS_MMDA13 with the members of the *Nipunavirus* genus NP1 and PaMx25 by using TBLASTX. 

The percentage of homologous proteins between vB_StuS_MMDA13 and the nearest phages has been assessed in pairwise comparison with CoreGenes v. 3.5 [58], while the ViPTree server v. 1.9 was employed to generate a protein-based phylogenetic tree (https://www.genome.jp/viptree/).

The nucleotide sequence of vB_StuS_MMDA13 was deposited in the GenBank database under accession number MN820898.

## 3. Results and Discussion

### 3.1. Phage Isolation and Morphological Features

**Figure 1:**
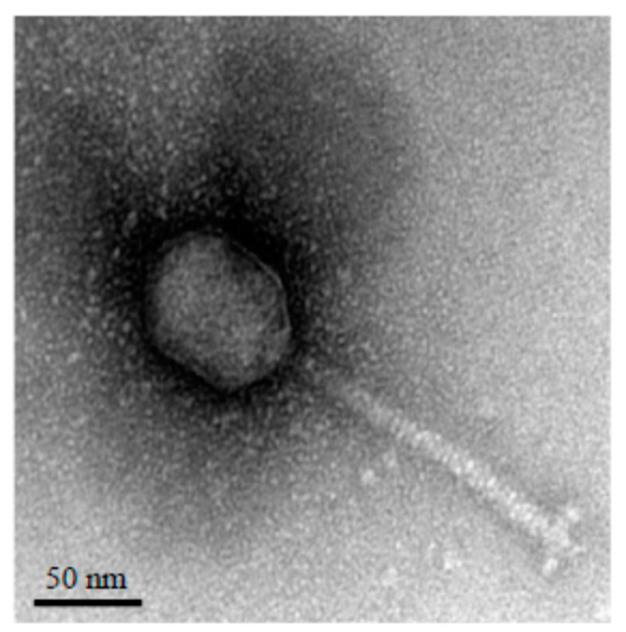
Transmission electron micrograph of the vB_StuS_MMDA13 phage negatively stained with uranyl acetate. The bar indicates 50 nm.

The vB_StuS_MMDA13 phage was isolated from a surface freshwater sample, collected during 2018 in a pond near Viterbo (Italy), by using *S. turrisvirgatae* MCT13^T^ as indicator strain. vB_StuS_MMDA13 forms small, clear plaques of about 1 mm diameter with regular edges. TEM analysis showed morphological features typical of the B2 morphotype of the *Siphoviridae* family members [59], with an icosahedral elongated head (≈70 × 55 nm) and a long, flexible, helical, non-contractile and striated tail of about 140 nm ending with an apparent baseplate connected to short terminal fibers (Figure 1). 

To the best of our knowledge, vB_StuS_MMDA13 is the first characterized phage belonging to the *Siphoviridae* family known to infect members of the *Sphingomonas* genus. 

### 3.2. Host-Range Determination

A total of 21 strains, including eight *Sphingomonas* type strains and six strains belonging to the *Sphingomonadaceae* family, were used to study the host range of the vB_StuS_MMDA13 phage (Table 1). The results from these experiments demonstrated that the lytic spectrum of this phage is restricted to *S. turrisvirgatae* MCT13^T^. All the other tested strains, including the closest *S. turrisvirgatae* MCT13^T^ relative, *Sphingomonas koreensis* NBRC_16723, were insensitive to infection by vB_StuS_MMDA13, suggesting a narrow host-range of this phage. The scarcity of data on the host range of phages infecting *Sphingomonas* spp. did not allow us to evaluate if the observed narrow host range of vB_StuS_MMDA13 is a particular feature of this phage.

### 3.3. One-Step Growth Curve

**Figure 2:**
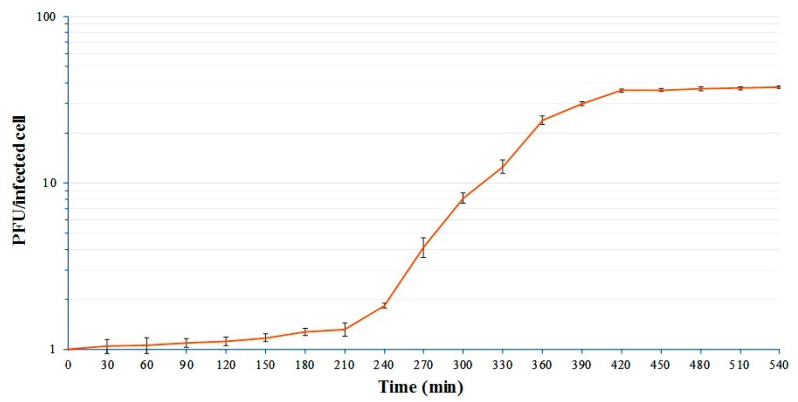
One-step growth curve of the vB_StuS_MMDA13 phage. Ratios between PFU and the number of infected bacterial cells at different time points are shown. Data are the mean of three independent experiments. Vertical black bars represent one standard deviation.

The one-step growth curve of vB_StuS_MMDA13 on *S. turrisvirgatae* MCT13^T^ (Figure 2) showed a long latency period of 210 min, followed by a rise period of about 200 min. These unexpectedly long latency and rise periods, previously observed also for other *Siphoviridae* infecting *Alphaproteobacteria* [22,24,60], could be related to the slow growth of the host bacteria in liquid media (estimated generation time of ≈2 h). The computed average burst size was about 30 PFU per infected cell. 

### 3.4. Phage Stability to pH and Temperature

The vB_StuS_MMDA13 phage exhibited a high stability to pH variations ranging from 3.0 to 11.0 after 1 h of incubation at 25 °C (Figure S1A). No massive reduction of phage infectivity was detected at pH 3.0, as just 1-log decrease of vB_StuS_MMDA13 titer was observed. At extreme acidic conditions, i.e., pH 2.0, the phage was completely inactivated. In the alkaline range, only the incubation at pH 12.0 caused approximately a > 1,000-fold drop of phage titer.

The effect of temperature on vB_StuS_MMDA13 activity was assessed by incubation at 25, 40, 50, 60, 70 and 80 °C. Results showed that vB_StuS_MMDA13 was highly stable in the range between 25 and 60 °C (Figure S1B), as no significant loss of infection ability was observed. Conversely, a decrease of approximately 100-fold in phage titer was observed after 60 min at 70 °C, while the phage was completely inactivated after 60 min at 80 °C.

### 3.5. Lysogeny Analysis

The induction with mitomycin C of three phage-insensitive colonies of *S. turrisvirgatae* MCT13^T^, obtained two days after a heavy infection, revealed no production of phage particles. A PCR screening, using two different primers pairs targeting vB_StuS_MMDA13 specific genes and DNA extractions from the same phage-insensitive derivatives, confirmed that the phage was not present in the *S. turrisvirgatae* MCT13^T^ genome (data not shown). Altogether, these findings demonstrate that vB_StuS_MMDA13 is lytic, and suggest that phage-insensitive derivatives are not susceptible to phage due to alterations in the bacterial structures required for phage infection. 

### 3.6. Bioinformatics Analysis of vB_StuS_MMDA13 Genome

High-throughput DNA sequencing of the vB_StuS_MMDA13 genome revealed that the phage is composed by a dsDNA of 63,743 bp characterized by a GC content of 59.3%. 

**Figure 3:**
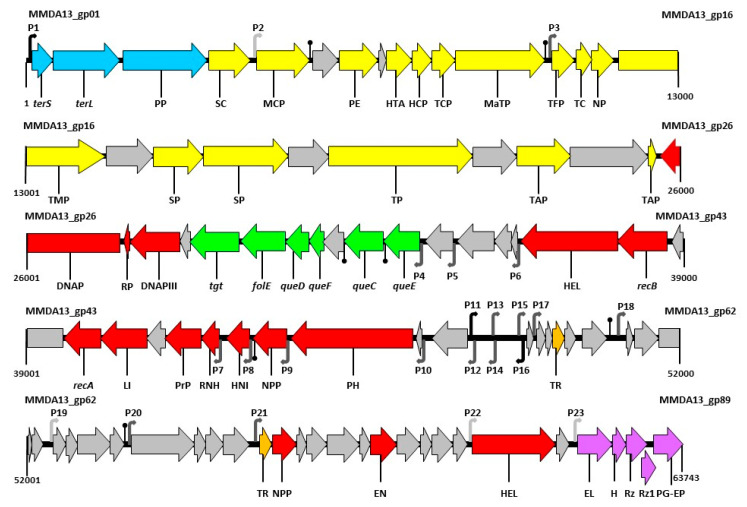
Functional genetic map of vB_StuS_MMDA13. Numbers indicate positions in the vB_StuS_MMDA13 genome (Accession number MN820898). Gene functions are assigned according to BLASTP and HHpred analyses. Colours of different functional modules are as follows: pale blue, packaging; yellow, morphogenesis; red, DNA replication/metabolism; green, modified nucleotides biosynthesis; orange, regulation; purple, lysis cassette. Open Reading Frames (ORFs) and a module of unknown function are reported in grey. ORFs names are as follows: *terS*, terminase small subunit; *terL*, terminase large subunit; PP, portal protein; SC, scaffold protein; MCP, major capsid protein; PE, peptidoglycan endopeptidase; HTA, head-tail adaptor; HCP, head-completion protein; TCP, tail completion protein; MaTP, main tail protein; TFP, tail fiber protein; TC, tail chaperonine; NP, neck protein; TMP, tail measure protein; SP, structural protein; TP, tail protein; TAP, tail assembly protein; DNAP, DNA polymerase; RP, DNA-directed RNA polymerase; DNAPIII, DNA polymerase III beta subunit; *tgt*, queuosine tRNA-ribosyltransferase; *folE*, GTP cyclohydrolase; *queD*, 6-carboxy-5,6,7,8-tetrahydropterin synthase; *queF*, 7-cyano-7-deazaguanine reductase; *queC*, 7-cyano-7-deazaguanine synthase; *queE*, 7-carboxy-7-deazaguanine synthase; HEL, helicase; *recB*, recB exonuclease; *recA*, recombinase A; LI, DNA ligase; PrP, primosomal protein; RNH, ribonuclease H; HNI, host-nuclease inhibitor; NPP, nucleotide pyrophosphohydrolase; PH, bifunctional primase helicase; TR, transcriptional regulator; EN, endonuclease; HEL, DNA helicase; EL, endolysin; H, holin/antiholin system; Rz, Rz protein; Rz1, Rz1 protein; PG-EP, peptidoglycan endopeptidase. Putative transcriptional promoters, detected with Bprom, are numbered (P1–P23) and shown as angled arrows according the transcription direction. The black, grey and pale grey arrows show putative promoters scoring more than 4, from 4 to 1, or less than 1, respectively. Putative rho-independent transcriptional terminators, detected with Genome2d, are depicted as vertical lines ending with a small black circle.

The result of genome assembly was circular, suggesting the presence of direct terminal repeats or circularly permuted terminal redundancy. However, a comparison with 86 terminases from phages with known packaging strategies was not able to predict a reliable packaging method, neither for vB_StuS_MMDA13 nor for members of the *Nipunavirus* genus [61] (data not shown). A total of 87 Open Reading Frames (ORFs) had been predicted by the RAST annotation service, and further manual inspection led to the identification of two additional ORFs (Supplementary Table S1). The coding percentage of the phage genome was equal to 94.3%, while the gene density was 1.40 gene/Kb. The majority of ORFs (76/89) have an ATG start codon, ten start with GTG, two with TTG and one with ATC. According to BLASTP, 24 (27.0%) proteins are similar to hypothetical proteins described in other phages, while 24 (27.0%) do not find significant matches or do not find matches at all. However, from HHpred and HMMER analyses a putative function can be assigned to 47 (52.8%) proteins (Supplementary Table S1). Sixty-one consecutive ORFs (38,382 bp; 60.2%) are encoded on the same DNA strand, while the remaining (21,208 bp; 33.3%) are on the opposite strand. These two regions are separated by a 1186 bp non-coding segment having a %GC of 50%, far lower than the rest of the genome, which is characterized by the presence of a 12 bp imperfect direct repeat (5′-T_3_G_2_C_4_G_4_T_4_T_4_A_4_A_4_C_4_C_4_A_3_T_4_-3′) present four times upstream of *gp54*. Six putative promoters, three for each DNA strand, have been also detected in this non-coding region. A total of 23 putative sigma70 promoters (Bprom) and seven putative rho-independent transcriptional terminators (Genome2d) were detected within the non-coding intergenic regions, with score values ranging from 0.25 to 4.54 and from 76 to 100, respectively (Figure 3). No tRNAs were found in the phage genome, suggesting that vB_StuS_MMDA13 cannot take over the host transcription/translation system, but relies on host tRNAs for the synthesis of phage proteins. This hypothesis is also supported by the observation that the same most abundant codon is shared between vB_StuS_MMDA13 and its host for 16/18 (88.9%) amino acids encoded by different triplets. Elements associated with the lysogenic replication cycle were absent.

The analysis with the VirFam tool suggests that vB_StuS_MMDA13 has a Neck Type 1 structural organization composed by a Portal (Gp3), an Adaptor (AD1, Gp9), a Head-Closure protein (HC1; Gp10), a type 1 Neck protein (Gp15) and a Type 1 Tail-Completion protein (TC1, Gp11). The genetic context of these deduced peptides and their relationship with the position of the terminase large subunit (Gp2), the major capsid protein (MCP, Gp5) and the main tail protein (MaTP, Gp12), suggest that vB_StuS_MMDA13 is a member of the VirFam Cluster 5, which is nearly exclusively composed of *Proteobacteria*-infecting phages. 

As commonly observed for the majority of bacteriophages, the vB_StuS_MMDA13 genome is organized in modules, with the genes related to different functions grouped in clusters. On the genome, the transcription directions diverge from the 1186 non-coding region interposed between *gp53* and *gp54* (Figure 3).

**Figure 4:**
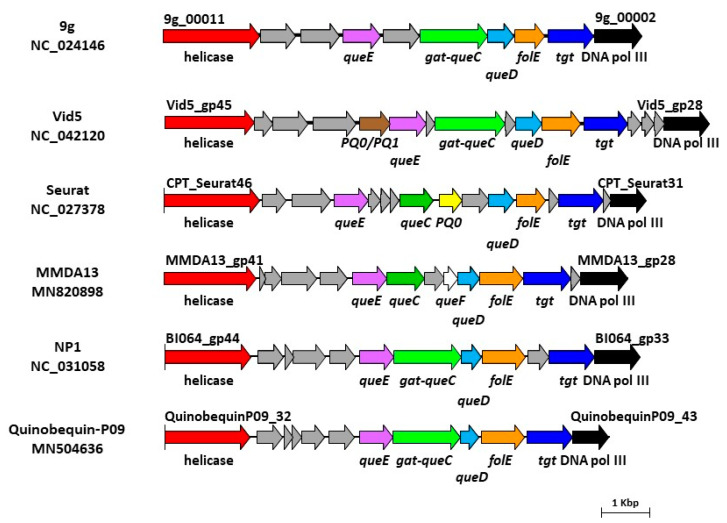
Gene clusters involved in the biosynthesis of 7-deazaguanine derivatives in vB_StuS_MMDA13, in type species of selected International Committee on Taxonomy of Viruses (ICTV) genera, and in the recently characterized Quinobequin-P09 phage. Functions of each deduced protein were assigned by HHpred analysis, by using a ≥90% probability cut-off. Abbreviations are as follows: DNA pol III, DNA polymerase III beta subunit; PQ0/PQ1, PreQ0/PreQ1 transporter; PQ0, PreQ0 transporter. Genes encoding hypothetical proteins are in grey.

The DNA replication/nucleotide metabolism module encompasses 28 ORFs and, from a BLASTP analysis, just five proteins (Gp34, Gp37, Gp38, Gp39 and Gp43) are homologous to hypothetical proteins of unknown functions described in other bacteriophage genomes. However, according to a HHpred analysis, all but two ORFs (*gp43* and *gp46*) encoded in the *gp41*-*gp51* region are predicted to be linked to replisome formation or nucleotide metabolism. In particular, this module includes a putative biosynthetic gene cluster (Figure 4) for the synthesis of modified nucleotides composed of six ORFs including homologues of a queuosine tRNA-ribosyltransferase (TGT, now called DpdA [62], Gp30), a GTP cyclohydrolase, (FolE, Gp31), 6-carboxy-5,6,7,8-tetrahydropterin synthase (QueD, Gp32), a 7-cyano-7-deazaguanine reductase (QueF-like, Gp33), a 7-cyano-7-deazaguanine synthase (QueC, Gp35) and an organic radical activating enzyme, (QueE, Gp36). According to a HHpred analysis Gp33 is predicted to be a QueF-like archaeosine synthase (91% probability). A further support of this result was obtained by comparing Gp33 with the two proteins QueF-L of *Aeropyrum pernix* K1 (BAA80469) and QueF of *Escherichia coli* (WP_000100421) used as anchors by Hutinet et al. [63] to search for the respective orthologues in bacteriophages. Interestingly, Gp35 (QueC) lacks the glutamine amido-transferase (GAT) domain found in other related phages, such as the 9g, NP1, Quinobequin-P09 and Vid5 [63,64,65,66], and shows the best BLASTP score with a putative queuosine biosynthesis protein (AGC35898) described in the *Myoviridae Rhizobium* phage RHEph06. Altogether these data suggest that vB_StuS_MMDA13 belongs to the first group proposed by Hutinet et al. [63], and that it is likely able to modify its own DNA with archaeosine. As depicted in Figure 4, therefore, the vB_StuS_MMDA13 nucleotide modification gene cluster differs from those of related viral genera by having a canonical QueC protein, with no GAT domain, and a separate amido-transferase function provided by the QueF-L homologue Gp33. 

The late genes, including those involved in the host lysis and the virion morphogenesis and packaging, are located downstream from the 1186 bp non-coding region, where a 12.7 Kbp segment including 31 ORFs mostly of unknown function precedes the host lysis cassette (Figure 3). Indeed, on the basis of BLASTP, HHpred or both, a putative function could be assigned only to 7/31 of these predicted proteins: Gp57 (repressor of the TetR/AcR family), Gp72 (anti-terminator, similar to the Lambda Q protein), Gp73 (nucleotide pyrophosphatase), Gp78 (Holliday Junction resolvase), Gp79 (thiol-dioxygenase), Gp81 (member of the ATP-binding cassette sub-family B) and Gp83 (UvrD DNA helicase).

The host lysis module of vB_StuS_MMDA13 included five ORFs (*gp85*–*gp89*) and is composed by: (i) an endolysin encoding gene (*gp85*) whose product shares 40% identity (coverage 71%) with the endolysin RL-2015 cloned from an *Acinetobacter* phages DNA pool [67]; (ii) a holin/antiholin system encoded by *gp86* and characterized by features typical of class II holins [68]; (iii) a Rz-like gene (*gp87*) whose deduced amino acid sequence showed a SPII cleavage site resulting in a typical proline rich (10%) mature protein [69] and (iv) a Rz1-like gene (*gp88*) which overlaps Rz.

The Rz/Rz1 system of vB_StuS_MMDA13, therefore, is of the “overlapped” type [69], and does not share significant similarities with other viral proteins in the databases. The lysis cassette ends with *gp89* whose protein product is recognized by HHpred as a peptidoglycan endopeptidase of the RipA–family, whose members are known to synergistically interact with Rpf(s) in the degradation of peptidoglycan [70]. 

Downstream from the lysis cassette three ORFs, *gp1*, *gp2* and *gp3*, form the packaging module encoding for the terminase small subunit, terminase large subunit and the portal protein, respectively. 

Finally, the morphogenesis module of vB_StuS_MMDA13 (*gp4*-*gp25*) starts with the head structural proteins (Gp4 and Gp5), followed by the adaptor (Gp9) and the head-completion protein (Gp10), and several proteins involved in the assembly of tail and neck (Gp11-Gp25). Interestingly, although the proteins involved in the head formation are more similar to homologues of *Nipunavirus*, those encoding tail components are more closely related to members of the *Septimatrevirus* or the *Lokivirus*. 

**Figure 5:**
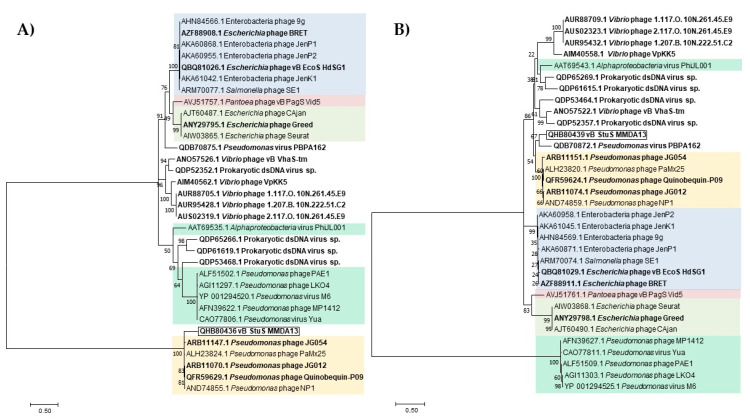
Maximum likelihood trees of terminase large subunits (**A**) and major capsid proteins (**B**). Trees were obtained with MEGA version X by using default parameters and 100 bootstraps. Bootstrap values are indicated next to tree branches. Protein accession numbers are reported for each branch. Colour scheme of different phage genera is as follows: pale blue, *Nonagvirus*; pale red, *Vidquintavirus*; pale green, *Seuratvirus*; green, *Yuavirus*; yellow, *Nipunavirus*. Bacteriophages not classified by ICTV (last accessed 30 July 2020) are in bold.

The taxonomic position of vB_StuS_MMDA13 has been tentatively evaluated by analyzing its terminase large subunit and major capsid protein sequences. In both cases the obtained phylogenetic trees gave results highly consistent with the current ICTV taxonomy, but also suggested that vB_StuS_MMDA13 could not be assigned to any of known genera (Figure 5). Indeed, even if in both cases vB_StuS_MMDA13 enters in the same clade with *Nipunavirus*, it forms distinct nodes. 

**Figure 6:**
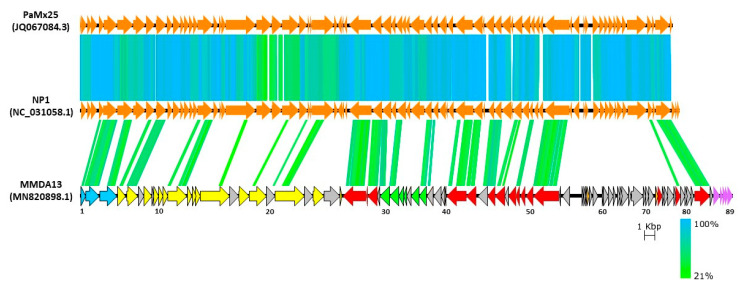
Comparison of vB_StuS_MMDA13 with the members of the *Nipunavirus* genus NP1 and PaMx25. ORFs are depicted according to the colouring scheme of Figure 3. Numbers indicate ORFs in the vB_StuS_MMDA13 genome. Homologues regions detected by a TBLASTX search are connected by segments coloured on the basis of amino acid identity. Homologues regions derived by segments <70 amino acids are not reported for clarity.

The remote relationship of vB_StuS_MMDA13 with viruses currently included in the *Nipunavirus* genus is also demonstrated by a TBLASTX analysis of the whole genome (Figure 6). 

**Figure 7:**
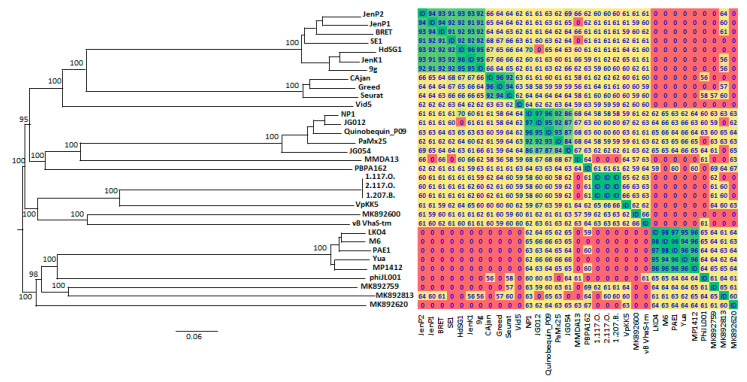
Phylogenomic Genome-BLAST Distance Phylogeny (GBDP) (on the left) and Orthologous Average Nucleotide Identity (OrthoANI) (on the right) analyses between vB_StuS_MMDA13 and the nearest members of *Siphoviridae* family. Only bootstrap values ≥ 95%, obtained by 100 replications in the GBDP analysis, are showed. Overall genomic sequence identity values are shown with different colours in a heatmap generated with the OrthoANI results. Cut-off is set at 50%: lower values are indicated as “0”. The following abbreviations are used: HdSG1, vB_EcoS_HdSG1; Vid5, vB_PagS_Vid5; MMDA13, vB_StuS_MMDA13; 1.117.O., Vibrio_phage_1.117.O._10N.261.45.E9; 2.117.O., Vibrio_phage_2.117.O._10N.261.45.E9; 1.207.B., Vibrio_phage_1.207.B._10N.222.51.C2; MK892600, Prokaryotic_dsDNA_virus_sp.; MK892759, Prokaryotic_dsDNA_virus_sp.; MK892813, Prokaryotic_dsDNA_virus_sp.; MK892620, Prokaryotic_dsDNA_virus_sp.

Moreover, very similar results were also obtained by analyzing the full genome with the Victor web-service, the OAT software (Figure 7) and the ViPTree web-service (Figure S2). According to the OAT analysis, vB_StuS_MMDA13 displayed a nucleotide identity ranging from 67% to 68% with the *Nipunavirus* classified by ICTV and with the unclassified phages JG012, JG054 and Quinobequin_P09. Interestingly, similar scores are also obtained when members of different genera are compared (e.g., Cajan and SE1, HdSG1 or JenK1; HdSG1 and NP1). On the other hand, the OAT proteome score with *Pseudomonas* phage PBPA 162 was lower (64%), although this phage yielded the best MCP homology and fell in the same node within the Victor tree. A CoreGenes analysis was performed to compare vB_StuS_MMDA13 with the type phages of the genera *Nipuna*-, *Nonag*-, *Seurat*-, *Vidquinta-virus* and with all the unclassified bacteriophages selected according to the MCP similarities. The results showed that the highest percentage of shared ORFs (42.7 to 43.8%) was observed with the *Nipunavirus*/Quinobequin-P09 group. Pairing of vB_StuS_MMDA13 *Pseudomonas* phage PBPA 162 yielded 37.1%.

## 4. Conclusions

In this work we characterized the first lytic *Siphoviridae* infecting members of the *Sphingomonas* genus. Despite that several bacteriophages able to lyse members of the *Sphingomonadaceae* family [23,26,27] have been described, very few provide an in-depth characterization [22,24,25]. Our results suggest that vB_StuS_MMDA13 does not belong to any known bacteriophage genera. Indeed, according to Adriaenssens et al. [71], a cut-off equal to 40% of shared proteins is the breakpoint to group two *Siphoviridae* in the same genus, providing there is consistency of other features such as genome size and organization, morphology, packaging and replication mechanisms. In the case of vB_StuS_MMDA13, the inclusion in one of the described genera should be only possible for the *Nipunavirus* genus. Indeed, the Victor analysis places vB_StuS_MMDA13 in a distant branch of the *Nipunavirus* (ICTV)/Unclassified *Nipunavirus*/Quinobequin_P09 clade, a result in agreement with the OAT analysis. The same branching is observed in the terminase tree, suggesting a common packaging strategy, and in the tree based on whole protein comparison obtained at the ViPTree website (Figure S2) [72]. There are, however, several differences between members of the *Nipunavirus* and vB_StuS_MMDA13: (i) the mean genome size of *Nipunavirus* (58.2 Kbp) is ≈5.5 Kbp smaller than that of our phage (63.7 Kbp), as are the ORFs number (74 vs. 89) and the GC content (mean of 58.5% for *Nipunavirus* vs. 59.3% of vB_StuS_MMDA13); (ii) *Nipunavirus* infect *Pseudomonas aeruginosa*, and the host range of vB_StuS_MMDA13 seems restricted to *S. turrisvirgatae*; (iii) the queuosine biosynthetic pathway is different, with vB_StuS_MMDA13 having a QueC rather than a GATase/QueC homologue, and a putative QueF-L which is lacking in *Nipunavirus*. Moreover, the whole host-lysis module is completely different: it shares no homologies, includes five components in vB_StuS_MMDA13 and four in *Nipunavirus*, and the putative holins belong to different classes. Finally, some important features of *Nipunavirus* are not present in vB_StuS_MMDA13, e.g., thymidylate synthase, DNA topoisomerase, and the ribonucleotide reductase of class II. We believe, therefore, that vB_StuS_MMDA13 should not be regarded as a distant species in the *Nipunavirus* genus but should rather represent the type strain of a newly discovered genus, which we propose to be named *Ememdadecimater-like virus*, after the short name of the first characterized phage of the genus.

## Figures and Tables

**Table 1:** Strains used for the determination of the vB_StuS_MMDA13 host range. +, lysed; −, not lysed.

Strain	Growth Medium ^a^	Susceptibility to vB_StuS_MMDA13	Reference
*Sphingomonas turrisvirgatae* MCT13^T^	ZBB	+	[28]
*Sphingomonas koreensis* NBRC_16723	ZBB	−	[31]
*Sphingomonas cynarae* DSM 25525	SPB	−	[32]
*Sphingomonas hankookensis* DSM 23329	ZBB	−	[33]
*Sphingomonas insulae* DSM 21792	ZBB	−	[34]
*Sphingomonas pseudosanguinis* DSM 19512	ZBB	−	[35]
*Sphingomonas panni* DSM 15761	ZBB	−	[36]
*Sphingomonas soli* DSM 18313	ZBB	−	[37]
*Sphingomonas naasensis* DSM 100060	ZBB	−	[38]
*Sphingobium yanoikuyae* DSM 7462	TSB	−	[39]
*Sphingobium chlorophenolicum* DSM 7098	NB	−	[39]
*Novosphingobium aromaticivorans* DSM 12444	PCA	−	[40]
*Novosphingobium capsulatum* DSM 30196	NB	−	[39]
*Sphingopyxis alaskensis* DSM 13593	TSB	−	[41]
*Sphingopyxis macrogoltabida* DSM 8826	NB	−	[39]
*Pseudomonas aeruginosa* PAO1	ZBB	−	[42]
*Pseudomonas aeruginosa* PAO4028	ZBB	−	-
*Pseudomonas aeruginosa* PA 661	ZBB	−	-
*Pseudomonas aeruginosa* ATCC 27853	ZBB	−	-
*Pseudomonas aeruginosa* ATCC 15442	ZBB	−	-
*Burkholderia cenocepacia* J2315 (CF5610)	ZBB	−	[43]
*Agrobacterium tumefaciens* DSM 5172	ZBB	−	-

^a^ Media abbreviations are as follows: ZBB, ZoBell Broth; SPB, SPhingo Broth (20 g/L glucose, 10 g/L peptone, 10 g/L yeast extract, 5 g/L NaCl); TSB, Tryptic Soy Broth; NB, Nutrient Broth; PCA, Plate Count Agar.

## Supplementary Materials

The following are available online at https://www.mdpi.com/1999-4915/12/8/894/s1, Figure S1: pH and temperature stability of vB_StuS_MMDA13, Figure S2: Proteomic phylogenetic tree of vB_StuS_MMDA13 and related phages, Table S1: General features of the coding regions of the vB_StuS_MMDA13 phage.
