# Supplementary material for: Characterization of vB_StuS_MMDA13, a Newly Discovered Bacteriophage Infecting the Agar-Degrading Species Sphingomonas turrisvirgatae
Source: Viruses. 2020 Aug 15;12(8):894. doi: 10.3390/v12080894 (PMC7472734; doi:10.3390/v12080894)
Supplement: Supplementary file 1 [file viruses-12-00894-s001.zip › viruses-732104_Figure S1 WL.pdf]

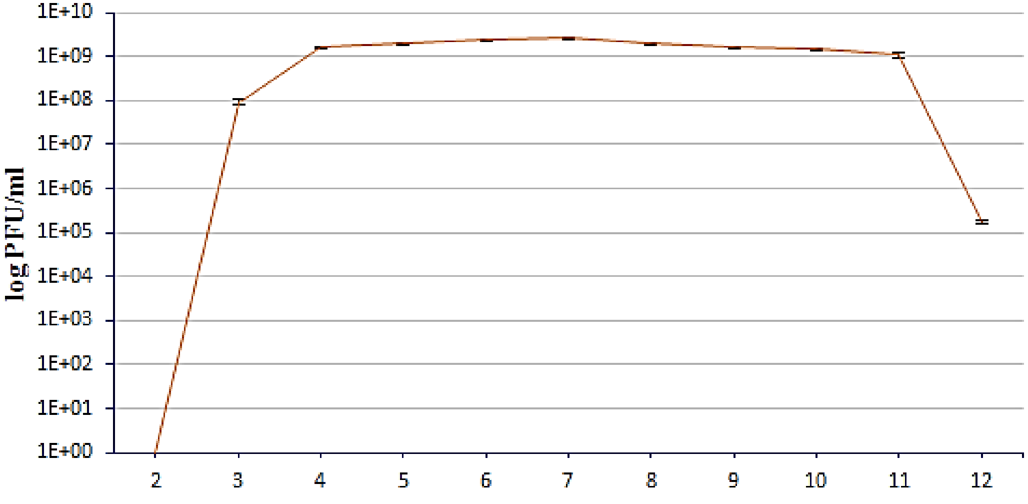

A)

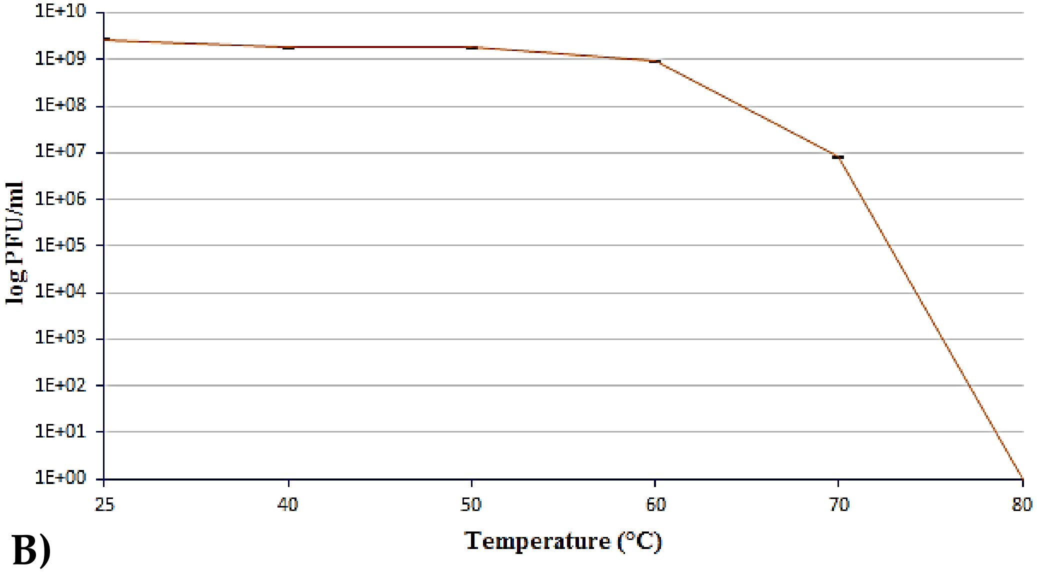

B)

Figure S1. pH **(A)** and temperature **(B)** stability of vB_StuS_MMDA13. Phages particles were incubated at 25 °C, in a pH range from 2.0 to 12.0 with intervals of 1 unit **(A)**, or at 25, 40, 50, 60, 70 and 80 °C **(B)**. In both cases incubation times were of 60 min, and data are the mean of three independent experiments. Vertical black bars represent one standard deviation.
