# Supplementary material for: Characterization of vB_StuS_MMDA13, a Newly Discovered Bacteriophage Infecting the Agar-Degrading Species Sphingomonas turrisvirgatae
Source: Viruses. 2020 Aug 15;12(8):894. doi: 10.3390/v12080894 (PMC7472734; doi:10.3390/v12080894)
Supplement: Supplementary file 1 [file viruses-12-00894-s001.zip › viruses-732104_Figure S2 WL.pdf]

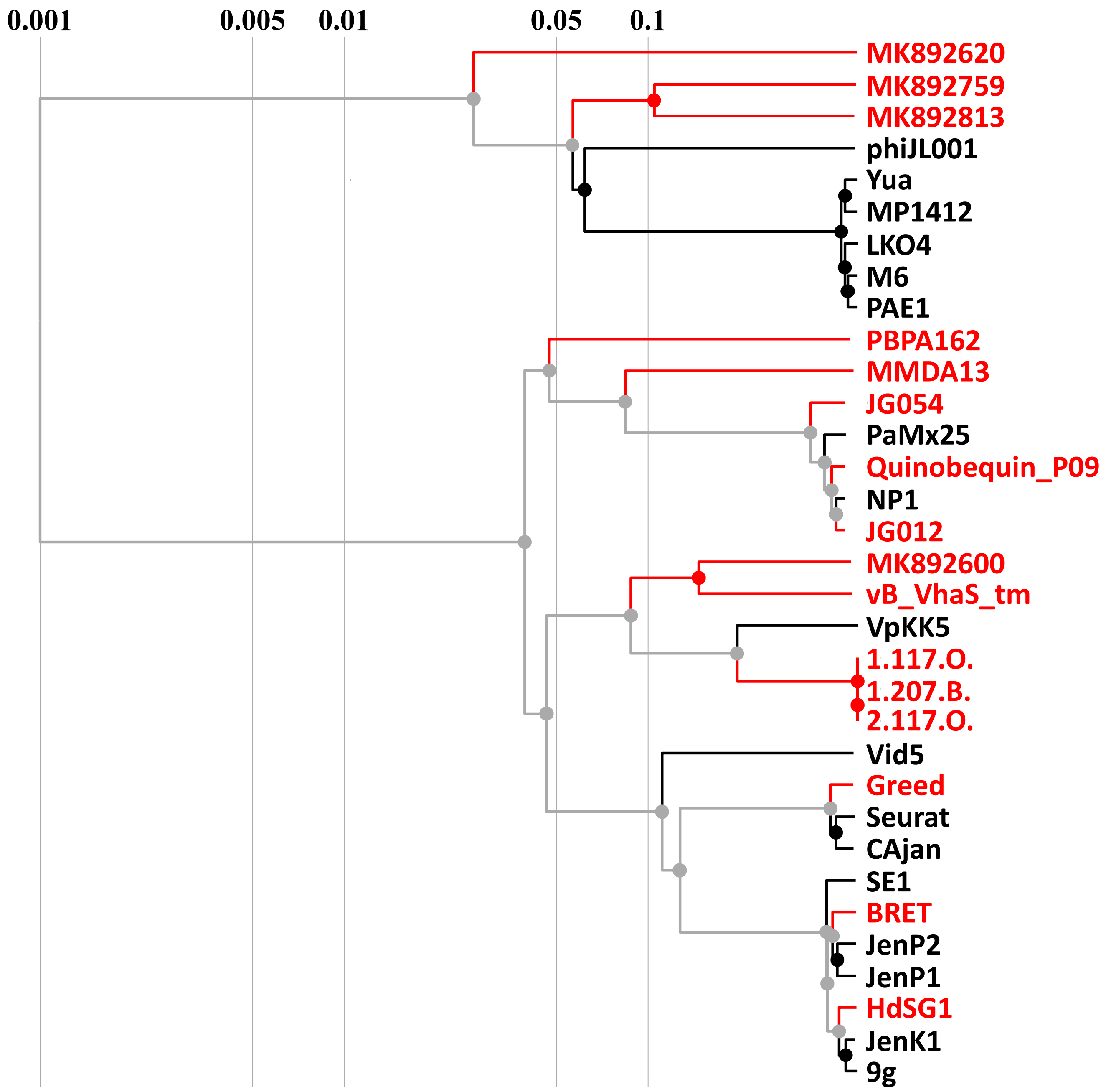

Figure S2. Proteomic phylogenetic tree based on a genome-wide similarity of viral genomes computed by TBLASTX and obtained by ViPTree web-server analysis. Branches and phage names of bacteriophages currently classified by ICTV (last accessed on 30 July 2020) are coloured in black.
