# Supplementary material for: Characterization of vB_StuS_MMDA13, a Newly Discovered Bacteriophage Infecting the Agar-Degrading Species Sphingomonas turrisvirgatae
Source: Viruses. 2020 Aug 15;12(8):894. doi: 10.3390/v12080894 (PMC7472734; doi:10.3390/v12080894)
Supplement: Supplementary file 1 [file viruses-12-00894-s001.zip › TAB S1 ORF MMDA13.pdf]

Table S1. General features of the coding regions of vB_StuS_MMDA13 phage. The closest homologues in INSDC databases, as resulted from a BLASTP search using "Viruses (taxid:10239)" as the organism filter, the possible conserved domains and the HHpred predicted motives are also shown. BLASTP hits of eukaryotic viruses were not taken into consideration.

gp	Start (nt	Stop (nt)	Predicted putative function	Closest protein homolog	Query overage (%)	ID (%)	Phage	Conserved domains (from-to; accession; short nam	HHpred motif of closes homologue	Motif definition	Probability (%
1	85	516	Terminase small subunit	ARB11146.1	95	43.97	*Pseudomonas* phage JG054	----------	3ZQP_H	Terminase small subunit	99.81
2	513	1847	Terminase large subunit	ALH23824.1	100	77.25	*Pseudomonas* phage PaMx25	40-237; cl29365; Terminase_6 super family	5OE8_C	Large subunit terminase	100
3	1908	3587	Portal protein	ARB11148.1	91	57.45	*Pseudomonas* phage JG054	312-448; cl16196; DUF4055 super family	2JES_G	Unidentified fragment of portal protein	99.9
4	3625	4443	Head protein	ARB11073.1	100	50.55	*Pseudomonas* phage JG012	----------	6B0X_c	Major head protein, Scaffold protein	94.97
5	4566	5630	Major phage capsid protein	ALH23820.1	100	71.75	*Pseudomonas* phage PaMx25	172-263; cl27082; Phage_capsid super family	6R3A_C	Major capsid protein	100
6	5690	6208	Hypothetical protein	ALH23819.1	100	53.18	*Pseudomonas* phage PaMx25	----------	5FVD_B	Nucleocapsid Phosphoprotein	20.43
7	6220	7002	Peptidoglycan endopeptidase domain protein	ARB11153.1	96	34.77	*Pseudomonas* phage JG054	----------	4Q4G_X	Peptidoglycan endopeptidase RipA	93.73
8	7007	7168	Hypothetical protein	QDP58552.1	45	58.33	Prokaryotic dsDNA virus sp.	----------	1K1F_E	Breakpoint cluster region protein	70.16
9	7172	7672	Head-tail adaptor	ALH23816.1	100	45.98	*Pseudomonas* phage PaMx25	----------	1XN8_A	Hypothetical protein yqbG	97.54
10	7672	8076	Head completion protein	QDB70185.1	34	39.13	*Salmonella* virus KFS-SE2	----------	1K0H_A	gpFII	96.49
11	8073	8540	Tail completion protein	ALH23585.1	94	34.25	*Pseudomonas* phage PaMx42	----------	2L25_A	Uncharacterized protein	99.91
12	8546	10387	Major tail protein	AXC34265.1	85	48.22	*Escherichia* phage Halfdan	----------	5NGJ_B	Tail tube protein	99.47
13	10463	10906	Tail fiber protein	QDP56586.1	100	48.30	Prokaryotic dsDNA virus sp.	----------	2OB9_A	Tail assembly chaperone	94.94
14	10918	11283	Tail chaperonin	AXC34263.1	90	44.07	*Escherichia phage* Halfdan	----------	5Z2H_A	*Dictyostelium discoideum* mitochondrial calcium uniporter	50.19
15	11252	11695	Head to tail connection protein (Neck)	CUS06475.1	95	42.55	*Acinetobacter* phage vB_AbaS_Loki	7-56; pfam04883; HK97-gp10_like	4N3Y_A	Rab5 GDP/GTP exchange factor, Rab	34.75
16	11699	14569	Phage tail tape measure protein	AEX56104.1	63	29.13	*Burkholderia* phage KL1	214-320; cl06039; TMP_2 super family	5IJO_T	Nuclear pore complex protein Nup155	94.67

17	14574	15512	Hypothetical protein	AEX56075.1	99	31.23	*Burkholderia* phage KL1	----------	1J3M_B	The conserved hypothetical protein TT1751	48.86
18	15512	16504	Structural phage protein	CAO77826.1	97	26.91	*Pseudomonas* virus Yua	----------	1J3M_B	The conserved hypothetical protein TT1751	57.29
19	16504	18198	Distal tail protein	AEX56105.1	94	31.29	*Burkholderia* phage KL1	----------	6F2M_C	Distal tail protein	96.5
20	18195	19004	Hypothetical protein	AEX56106.1	100	41.39	*Burkholderia* phage KL1	178-262; cl10710; Phage_BR0599 super family) 31-268; cl37077; phg_TIGR02218 super family	6J0M_C	Pvc8	51.19
21	19004	21859	Tail protein	AEX56107.1	82	39.20	*Burkholderia* phage KL1	----------	3CDD_E	Prophage MuSo2, 43 kDa tail	96.28
22	21856	22746	Hypothetical protein	AGH31476.1	37	30.00	*Loktanella* phage pCB2051-A	----------	4OUS_A	Caprin-2	67.21
23	22746	23798	Tail assembly protein	ATS92205.1	99	31.84	*Stenotrophomonas* phage DLP4	----------	2HB0_A	CFA/I fimbrial subunit E	20.75
24	23795	25357	Hypothetical protein	AEY69562.1	15	38.55	*Burkholderia* phage vB_BceS_AH2	----------	4UWA_D	Ryanodine receptor 1	99.85
25	25357	25521	Tail assembly protein	AHB12066.1	94	37.25	*Xylella* phage Sano	----------	2QZB_B	Uncharacterized protein yfeY	25.4
26	27851	25626	DNA polymerase	ARB11098.1	98	61.41	*Pseudomonas* phage JG012	----------	5MDN_B	DNA polymerase (E,C,2,7,7, 7)	100
27	28034	27903	DNA-directed RNA polymerase	AUR82457.1	60	46.15	*Vibrio* phage 1.024.O._10N.261.45.F8	----------	4QIW_W	DNA-directed RNA polymerase (E,C,2,7,7,6)	98.49
28	29026	28034	DNA polymerase III beta subunit	ARB11176.1	96	58.81	*Pseudomonas* phage JG054	----------	3T0P_B	DNA polymerase III, beta subunit	100
29	29226	29020	Hypothetical protein	No match				----------	2CYU_A	2-oxoglutarate dehydrogenase multienzyme complex (E,C,2,3,1,61)	59.88
30	30200	29223	Queuosine tRNA ribosyltransferase (DpdA)	AND74884.1	99	70.06	*Pseudomonas* phage NP1	----------	2ASH_D	Queuine tRNA-ribosyltransferase (E,C,2,4,2,29)	100
31	31106	30219	GTP cyclohydrolase I (FolE)	AND74886.1	89	60.37	*Pseudomonas* phage NP1	----------	4UQF_C	GTP cyclohydrolase 1 (E,C,3,5,4,16)	100
32	31573	31106	6-carboxytetrahydropterin synthase (QueD-like)	AGC35897.1	96	52.35	*Rhizobium* phage RHEph06	----------	2G64_A	Putative 6-pyruvoyl tetrahydrobiopterin synthase (E.C.4.2.3.12)	100
33	31872	31576	Archeaosine synthase QueF-Like	QDP52302.1	92	43.96	Prokaryotic dsDNA virus sp.	----------	5K0P_G	Archeaosine synthase QueF-Like	91.16
34	32261	31848	Hypothetical protein	QDP52303.1	91	46.92	Prokaryotic dsDNA virus sp.	----------	6OHT_A	3-beta-hydroxysteroid-Delta(8), Delta(7)-isomerase (E,C,5,3,3,5)	11.85

35	33046	32261	7-cyano-7-deazaguanine synthase (QueC-like)	AGC35898.1	96	56.42	*Rhizobium* phage RHEph06	----------	3BL5_F	Queuosine biosynthesis protein queC (E,C,3,5,-,-)	99.93
36	33759	33046	7-carboxy-7-deazaguanine synthase (QueE-like)	ALH23789.1	99	53.36	*Pseudomonas* phage PaMx25	----------	4NJH_B	7-carboxy-7-deazaguanine synthase (E,C,4,3,99,3)	99.92
37	34428	33868	Hypothetical protein	ALH23788.1	52	73.20	*Pseudomonas* phage PaMx25	----------	3JCJ_f	Protein/RNA Complex	93.94
38	35227	34505	Hypothetical protein	ARB11183.1	100	42.69	*Pseudomonas* phage JG054	----------	2NP3_B	Putative TetR-family regulator	81.33
39	35579	35229	Hypothetical protein	QDP53518.1	50	41.67	Prokaryotic dsDNA virus sp.	----------	5W1S_N	DNA-directed RNA polymerase subunit alpha	81.09
40	35697	35569	Hypothetical protein	No match				----------	4D7S_B	STHK_CNBD_CGMP	68.16
41	37681	35756	Superfamily II DNA helicase	ARB11186.1	96	58.27	*Pseudomonas* phage JG054	----------	6JDE_A	Putative DNA repair helicase RadD	100
42	38649	37678	Exonuclease RecB	ARB11187.1	97	58.82	*Pseudomonas* phage JG054	----------	3SYY_A	Exonuclease	99.3
43	39739	38774	Hypothetical protein	AND74897.1	52	65.70	*Pseudomonas* phage NP1	----------	1NY4_A	30S ribosomal protein S28E	51.51
44	40497	39772	RecA ATPase	AND74898.1	100	75.10	*Pseudomonas* phage NP1	----------	1N0W_A	DNA repair protein RAD51 homolog	99.41
45	41435	40497	ATP-dependent DNA ligase	QDB70915.1	97	45.63	*Pseudomonas* virus PBPA162	----------	1FVI_A	PBCV-1 DNA ligase (E,C,6,5,1,-)	100
46	41809	41432	Hypothetical protein	No match				----------			
47	42509	41784	Primosomal protein	QFR59661.1	75	39.89	*Pseudomonas* phage Quinobequin-P09	----------	2D7E_B	Primosomal protein N'(E,C,3,6,1,-)	92.76
48	42865	42506	Ribonuclease H like protein	ARB11119.1	98	45.30	*Pseudomonas* phage JG012	----------	4IBN_A	Ribonuclease H (E,C,3,1,26,4)	98.89
49	43431	42973	Host-nuclease inhibitor	ALH23774.1	92	43.97	*Pseudomonas* phage PaMx25	----------	2P2U_A	Host-nuclease inhibitor protein Gam, putative	95.55
50	44179	43499	Nucleotide pyrophosphohydrolase	ARK07441.1	82	39.04	*Sphingobium* phage Lacusarx	----------	4QGP_B	Pyrophosphatase	98.92
51	46673	44244	Primase	ARB11197.1	99	56.01	*Pseudomonas* phage JG054	----------	6A9W_A	Primase	100
52	46869	46735	Hypothetical protein	No match				----------	6Q6G_O	Cell division cycle protein 20	50.9
53	47759	47061	Hypothetical protein	No match				----------	1P2X_A	Ras GTPase-activating-like protein	62.77
54	48946	49089	Hypothetical protein	No match				----------	6OD1_A	Regulator of RpoS, Anti-adapter protein	52.13
55	49140	49322	Hypothetical protein	No match				----------	2N7R_A	Nicastrin	66.19
56	49324	49458	Hypothetical protein	No match				----------	6HIZ_DE	Uncharacterized protein, uS3m, UNK/RNA Complex	84
57	49463	49711	Transcriptional regulator	No match				----------	4ZA6_A	QsdR	95.48

58	49708	49920	Hypothetical protein	No match				----------	4AYB_A	DNA-directed RNA polymerase (E,C,2,7,7,6)	43.24
59	50046	50546	Hypothetical protein	ARB11185.1	97	45.78	*Pseudomonas* phage JG054	----------	2OUT_A	Mu-like prophage FluMu protein gp35	93.19
60	50923	51063	Hypothetical protein	No match				----------	2XJY_B	Rhombotin-2, LIM domain-binding protein 1	55.47
61	51090	51563	Hypothetical protein	No match				----------	4UC0_A	Purine nucleoside phosphorylase	53.72
62	51563	52039	Hypothetical protein	AEX55965.1	32	43.64	*Pseudomonas* virus PMG1	----------	6GAW_B5	Mitochondrial ribosomal protein L12, Mitochondrial	78.86
63	52039	52251	Hypothetical protein	QDP53750.1	68	50.00	Prokaryotic dsDNA virus sp.	----------	1QHK_A	Ribonuclease HI (E,C,3,1,26,4)	74.4
64	52447	52668	Hypothetical protein	No match				----------	6K15_E	RSC7, Chromatin structure-remodeling complex protein	51.94
65	52665	52892	Hypothetical protein	No match				----------	3JXO_A	TrkA-N domain protein	75.14
66	52879	53490	Hypothetical protein	QAX97590.1	95	33.58	*Enterococcus* phage EfsSzw-1	----------	6SGA_Fd	Mitochondrial ribosome, assembly intermediate, translation	54.23
67	53493	53759	Hypothetical protein	No match				----------	6MCJ_A	Putative orange carotenoid-binding protein	49.93
68	53832	54968	Hypothetical protein	ASE99804.1	42	31.52	Uncultured virus	----------	6DM9_B	DHD127_A, DHD127_B	50.34
69	54965	55180	Hypothetical protein	ARM66686.1	67	39.58	*Lactococcus* phage AM4	----------	3M9D_I	Coil coil domain, inter domain	49.13
70	55167	55505	Hypothetical protein	QBQ72133.1	57	35.38	*Brevundimonas* phage vB_BsubS-Delta	----------	1Y7T_A	Malate dehydrogenase (E,C,1,1,1,37)	41.86
71	55483	55962	Hypothetical protein	No match				----------	4OTN_A	Eukaryotic translation initiation factor 2-alpha	65.08
72	56139	56351	Antitermination protein	VCU43734.1	72	29.09	*Escherichia* virus vB_Eco_mar003J3	----------	4MO1_A	Antitermination protein Q	96.92
73	56360	56806	Nucleotide pyrophosphohydrolase	BBA65581.1	83	51.13	*Xanthomonas* phage XacN1	----------	4QGP_B	Pyrophosphatase	99.15
74	56803	56982	Hypothetical protein	No match				----------	6G2J_S	NADH-ubiquinone oxidoreductase chain 3 (E,C,1,6,5,3)	48.47
75	56975	57358	Hypothetical protein	QDP54416.1	51	47.69	Prokaryotic dsDNA virus sp.	----------	2O35_B	Hypothetical protein DUF1244	47.95

76	57358	57936	Hypothetical protein	No match				----------	6QZL_D	FAM83B	74.8
77	57933	58133	Hypothetical protein	ADU79191.1	39	38.46	*Enterobacter* phage EcP1	----------	3M05_A	Uncharacterized protein PEPE_1480	87.21
78	58133	58606	Endonuclease	QDB70880.1	98	43.75	*Pseudomonas* virus PBPA162	----------	1OB8_B	Holliday-junction resolvase	99.3
79	58606	59034	Thiol dioxygenase	AID18361.1	66	53.19	*Rhizobium* phage vB_RleM_PPF1	----------	4WVZ_B	Thiol dioxygenase	97.33
80	59036	59239	Hypothetical protein	No match				----------	1IIO_A	Conserved hypothetical protein MTH865	56.23
81	59229	59615	Hypothetical protein	No match				----------	3NH6_A	ATP-binding cassette sub-family B member	98.43
82	59612	59875	Hypothetical protein	ALY08076.1	37	39.39	*Bacillus* phage vB_BhaS-171	----------	5IJO_T	Nuclear pore complex protein Nup155	51.14
83	59945	61462	UvrD superfamily I DNA helicase	ARB11211.1	100	62.57	*Pseudomonas* phage JG054	----------	4C2T_D	DNA helicase II (E,C,3,6,4,12)	100
84	61473	61691	Hypothetical protein	No match				----------	3MSH_A	Hepatitis B virus X-interacting protein	55.43
85	61849	62481	Endolysin	AJG41882.1	71	39.74	*Acinetobacter* phage RL-2015	----------	3NE0_A	Resuscitation promoting factor Interacting Protein	98.85
86	62478	62732	Holin	APZ81838.1	86	87.67	*Erythrobacter* phage vB_EliS_R6L	----------	2O6K_B	UPF0346 protein MW1311	63.93
87	62725	63105	Rz protein	No match				----------	5LQZ_V	ATP synthase subunit f, ATP	93.62
88	62993	63283	Rz1 protein	No match				----------	4IMM_B	Outer membrane assembly lipoprotein YfgL	91.68
89	63213	63743	Peptidoglycan endopeptidase	No match				----------	4Q4G_X	Peptidoglycan endopeptidase RipA	90.83
